# Dual checkpoint blockade of PD-1 and Tim-3 by engineered hybrid nanovesicles for enhanced cancer immunotherapy

**DOI:** 10.3389/fimmu.2025.1705438

**Published:** 2025-12-17

**Authors:** Han Xue, Longxue Guan, Lili Huang, Yuxin Fan, Fenglin Guo, Dandan Liang, Xingang Guan, Guofu Chen

**Affiliations:** 1 The First People’s Hospital of Wenling (Taizhou University Affiliated Wenling Hospital), School of Medicine, Taizhou University, Taizhou, China; 2 College of Medical Technology, Beihua University, Jilin, China

**Keywords:** PD-1, Tim-3, cell membrane nanovesicle, antitumor immunity, dual checkpoint blockade

## Abstract

T cell immunoglobulin and mucin domain-containing protein 3 (Tim-3) is an inhibitory receptor expressed on immune cells, and its co-expression with programmed cell death protein 1 (PD-1) is an established mechanism of immune exhaustion and resistance to checkpoint blockade. To overcome this, we developed PD-1/Tim-3-decorated nanovesicles (PD-1/Tim-3 NVs) for combination immunotherapy against colorectal cancer. These vesicles selectively engaged the ligands PD-L1 and Galectin-9. In mice bearing CT26 xenografts, PD-1/Tim-3 NVs suppressed tumor growth by 69.0%, remodeled the tumor microenvironment by enhancing CD8^+^ T cell infiltration and activation, and depleting immunosuppressive regulatory T cells. Our findings highlight the promising potential of simultaneous PD-1 and Tim-3 blockade for treating advanced tumors.

## 1 Introduction

Cancer immunotherapy has demonstrated unprecedented efficacy in treating a wide range of advanced malignancies over the past decade (1, 2). Immune checkpoint inhibitors (ICIs) have arisen as among the most potent of these immunotherapies, capable of eliciting durable and systemic antitumor immune responses in cancers such as melanoma and non-small cell lung cancer (NSCLC) (3–5). Despite this success, their clinical application is significantly limited by the low overall response rate, primary and acquired resistance, and immune-related adverse events (irAEs) (6, 7). To overcome this limitation, combination strategies that pair ICIs with other treatment modalities (e.g., chemotherapy, radiotherapy, targeted therapy, or immunotherapy) have substantially improved the therapeutic outcomes in many advanced cancers (8–11).

T cell immunoglobulin and mucin domain-containing protein 3 (Tim-3, also known as HAVCR2), a member of the T cell immunoglobulin and mucin domain protein family, is an immune checkpoint receptor expressed on monocytes, dendritic cells (DCs), and natural killer (NK) cells (12, 13). Like PD-1, Tim-3 functions as an inhibitory receptor that suppresses immune responses in both innate and adaptive immunity, and Tim-3 blockade promotes tumor regression and antitumor immune memory (14). As high Tim-3 expression is a hallmark of exhausted T cells, it has emerged as a promising target for cancer immunotherapy (15). Notably, abundant co-expression of Tim-3, LAG-3, and PD-1 is frequently observed in advanced cancers, where these receptors collaboratively contribute to clinical resistance against PD-1/PD-L1 blockade therapy (16–19). Given the pivotal roles of Tim-3 and PD-1 in suppressing antitumor immunity, concurrent blockade of both pathways represents a promising strategy to enhance response rates and overcome resistance in cancer patients who exhibit limited benefit from anti-PD-1/PD-L1 monotherapy (20, 21). To this end, therapeutic approaches such as combination checkpoint inhibition (anti-PD-1/PD-L1 plus anti–Tim-3), novel bispecific antibodies, or dual-targeting agents have emerged as critical avenues for improving the efficacy of cancer immunotherapy (22–24).

Drug delivery systems (DDSs) can enhance therapeutic efficacy by improving drug solubility, prolonging systemic circulation half-life, enabling stimuli-responsive drug release, and promoting cellular uptake (25, 26). As a class of nanocarriers derived from natural sources, cell membrane-derived nanovesicles have garnered significant attention owing to their high biocompatibility, biodegradability, favorable pharmacokinetic profiles, and ease of modification through genetic engineering (27–29). In addition to encapsulating various drugs or nanoparticles in the inner cavity, nanovesicles can also display functional proteins or peptides on their outer surface in their native conformation, endowing them with tumor targeting, antigen presentation, immune modulation, and other functionalities (30–33).

**Scheme 1:**
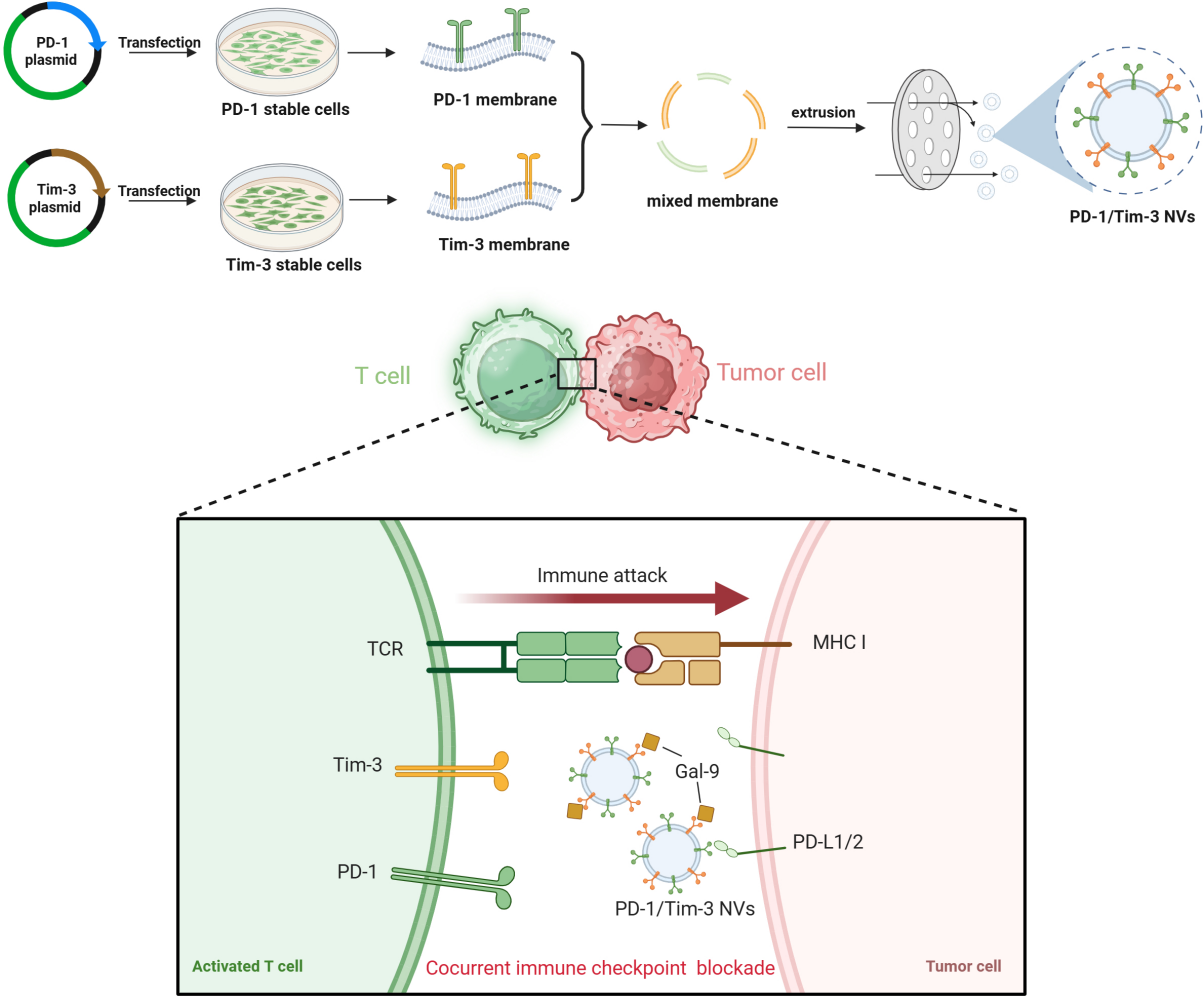
Schematic illustration of preparation and mechanisms for combined immunotherapy through dual checkpoint blockade.

Given the clinical success of combining anti-PD-1/PD-L1 therapy with other checkpoint inhibitors (e.g., anti-CTLA-4, anti-LAG-3) (34, 35), the development of novel formulations for concurrent immune checkpoint blockade is a promising strategy for improving therapeutic outcomes in advanced cancers. In this study, we developed a PD-1/Tim-3-decorated cell membrane nanovesicle (PD-1/Tim-3 NVs) for treating colorectal cancer (**Scheme 1**). The hybrid nanovesicles demonstrated selective binding to PD-L1 and galectin-9 (the ligand for Tim-3) on CT26 tumor cells. In murine tumor models, PD-1/Tim-3 nanovesicles (NVs) exhibited enhanced tumor accumulation and significantly suppressed tumor growth without inducing observable toxicity. This therapeutic effect was accompanied by increased infiltration and activation of CD8^+^ T cells, along with a marked reduction in regulatory T cell (Treg) populations, collectively driving a robust antitumor immune response. Our findings underscore the considerable potential of cell membrane nanovesicles for concurrent immune checkpoint blockade and establish a promising platform for next-generation combination immunotherapy.

## 2 Materials and methods

### 2.1 Materials

The lentiviral vector pLenti-C-PD-1-mGFP carrying the mouse PD-1 gene was obtained from OriGene Inc (CAT: MR227347L4). The Tim-3 expression plasmid pEZ-Lv130-Tim-3-mCherry was purchased from GeneCopoeia™ Inc (CAT: Mm30525). Lipofectamine™ 3000 was purchased from Thermo (CAT: L3000001). Cell membrane fluorescent probe DiO (CAT: C1038) and Dil (CAT: C1036), the nuclear staining dye DAPI (CAT: C1006), the BCA assay kit (CAT: P0009), and Cell Counting Kit-8 (CCK-8) (CAT: C0038), were acquired from Beyotime Biotechnology (China). Dulbecco’s Modified Eagle Medium (DMEM) (CAT: BL301A) and fetal bovine serum (FBS) (CAT: BL201A) were sourced from Biosharp (China). Antibodies for flow cytometry were procured from BioLegend (USA). PD-L1 antibodies for blockade assays were purchased from BioLegend (Clone: W20151E), and Galectin-9 (Gal-9) antibodies were obtained from Abclonal (CAT: A2516).

### 2.2 Cell lines

The mouse colorectal cancer cell line CT26 and the human embryonic kidney cell line HEK-293T were obtained from the American Type Culture Collection (ATCC) and cultured according to the provider’s recommended protocols. CT26 and HEK-293T cells were cultured in DMEM containing 10% FBS in a cell incubator at 37°C and 5% CO_2_.

### 2.3 Preparation of PD-1 or Tim-3 stable cell lines

HEK-293T cells were transfected with lentiviral vectors encoding PD-1–GFP or Tim-3–mCherry using Lipofectamine™ 3000. After transfection, the cells were cultured under selection with puromycin (5 μg/mL). Single-cell colonies were isolated via the limited dilution method to generate clonally derived stable lines. To verify membrane localization of PD-1-GFP and Tim-3-mCherry in the engineered HEK-293T cells, confocal laser scanning microscopy (CLSM) (Zeiss, LSM710) was performed using the membrane dyes Dil (red) and DiO (green).

### 2.4 Preparation and characterization of PD-1/Tim-3 NVs

PD-1 NVs and Tim-3 NVs were prepared by extruding plasma membranes derived from PD-1 or Tim-3-expressing stable cell lines, respectively. PD-1/Tim-3 NVs were fabricated by co-extruding a 1:1 (by protein weight) mixture of membranes isolated from PD-1 and Tim-3 stable cells. Briefly, cells were disrupted using a Dounce homogenizer on ice in homogenization buffer (20 mM HEPES-NaOH, pH 7.4, 0.25 M sucrose, 1 mM EDTA, 1 mM PMSF) with at least 50 strokes. The homogenate was subjected to ultracentrifugation at 35,000 rpm for 2 h to pellet crude membrane fractions. Membranes were washed with PBS and sonicated for 5 min. The membrane suspensions were then extruded through 1.0 μm and 0.4 μm polycarbonate membranes using a mini-extruder for a minimum of 20 passes. The resulting nanovesicles were designated as PD-1 NVs, Tim-3 NVs, or PD-1/Tim-3 NVs.

The size distribution of nanovesicles was measured at room temperature using a nanoparticle size and ζ potential meter (Microtrac, Nanotrac Wave II). The morphology of nanovesicles was applied to glow-discharged carbon-coated copper grids. The grids were fixed with 4% paraformaldehyde for 15 min and rinsed with deionized water. The grids were negatively stained with 2% uranyl acetate and applied with a JEM 1011 microscope (JEOL, Japan).

### 2.5 Western blotting analysis

The presence of PD-1 and Tim-3 in the nanovesicles was evaluated by Western blotting. Protein samples (20 µg per lane) were separated on 10% SDS-polyacrylamide gels and transferred to polyvinylidene fluoride (PVDF) membranes. The membranes were blocked with 5% (w/v) skim milk for 1 h at 25 °C, then incubated overnight at 4°C with primary antibodies specific to PD-1 or Tim-3. After washing, the membranes were probed with horseradish peroxidase (HRP)-conjugated secondary antibodies. Protein bands were visualized using a Chemiluminescent Substrate kit.

### 2.6 *In vitro* biocompatibility analysis

The biocompatibility of PD-1/Tim-3 NVs was evaluated using a CCK-8 assay to measure cell viability. HEK-293T cells were seeded in 96-well plates at a density of 5,000 cells per well and allowed to adhere overnight. Various concentrations of PD-1/Tim-3 NVs were then added to the wells, and the cells were incubated for 48 hours. After treatment, CCK-8 solution (5 mg/mL in PBS) was added to each well and incubated for 2 hours at 37°C. Absorbance was measured at 450 nm using a TECAN M200 microplate reader to determine relative cell viability.

### 2.7 *In vivo* biodistribution analysis

To track the biodistribution of PD-1/Tim-3 nanovesicles (NVs) in tumors and healthy organs, we prepared Cy5.5-labeled Blank NVs and PD-1/Tim-3 NVs using DSPE-Cy5.5. Mice bearing CT26 tumors received a single intravenous injection (via tail vein) of either Cy5.5-labeled Blank NVs or PD-1/Tim-3 NVs (0.2 mg total protein per mouse). At 24 hours post-injection, tumors and major organs were harvested and imaged using an *In-Vivo* Multispectral Imaging System FX (Kodak, Japan). Fluorescence intensity was quantified using the accompanying imaging software.

### 2.8 *In vivo* antitumor analysis

All animal procedures were performed in compliance with the National Institutes of Health Guidelines for the Care and Use of Laboratory Animals and were approved by the Animal Ethics Committee of Taizhou University (Protocol number: TZXY- 2022-20221031). A mouse tumor model was established by subcutaneous injection of CT26 cells (1 × 10^6^ cells) into the right flank of BALB/c mice. When tumor volumes reached 50~80 mm³, the mice were randomly assigned to four groups: PBS, PD-1 NVs, Tim-3 NVs, and PD-1/Tim-3 NVs (n=6). Treatments were administered via intratumoral injection every three days for a total of five doses, each containing 200 μg of the respective nanovesicles per mouse. Tumor dimensions and body weight were monitored regularly throughout the study. Tumor volume (V) was calculated using the formula: V = d²×D/2, where d and D represent the shortest and the longest diameter, respectively. Mice will be excluded from analysis in cases of failed tumor engraftment, pretreatment tumor volumes exceeding ethical thresholds, or the development of severe health issues during treatment.

### 2.9 Flow cytometry

Tumor-infiltrated immune cells were analyzed by flow cytometry analysis. For immune cells preparation, excised tumors were minced and digested in DMEM supplemented with DNase I and collagenase IA. The resulting cell suspension was filtered through a 200-mesh strainer and centrifuged at 1000 rpm for 5 minutes. Cells were then stained using the Zombie Violet™ Fixable Viability Kit and stained with the respective antibodies. For intracellular staining of granzyme B (GZMB) and perforin, cells were surface-stained with anti-CD45 and anti-CD8a, then fixed and permeabilized using the Cyto-Fast™ Fix/Perm Buffer Set (BioLegend), followed by intracellular staining with anti-GZMB and anti-perforin. For regulatory T cell (Treg) analysis, cells were surface-stained with anti-CD45 and anti-CD4, then fixed and permeabilized using the True-Nuclear™ Transcription Factor Buffer Set (BioLegend), and subsequently stained intracellularly with anti-Foxp3 (PE; clone MF-14). All samples were analyzed on a CytoFLEX flow cytometer (Beckman Coulter), and data were acquired using CytoExpert software (Version 2.4).

### 2.10 Tissue section staining

Major organs (liver, spleen, kidney, heart, and lung) and tumor tissues were collected from mice following treatment with different formulations. Tissues were fixed, paraffin-embedded, and sectioned for hematoxylin and eosin (H&E) staining according to standard protocols.

### 2.11 Inflammatory factor assay

Serum levels of interferon-gamma (IFN-γ) and tumor necrosis factor-alpha (TNF-α) were measured in mice treated with PBS, PD-1 NVs, Tim-3 NVs, or PD-1/Tim-3 NVs using commercial ELISA kits (Kaiji, China) according to the manufacturer’s instructions.

### 2.12 Biosafety evaluation

Blood samples were collected from mice following treatment with PBS, PD-1 NVs, Tim-3 NVs, or PD-1/Tim-3 NVs. Serum levels of alanine aminotransferase (ALT), aspartate aminotransferase (AST), uric acid (UA), blood urea nitrogen (BUN), and creatinine (Crea) were quantified using an Olympus AU2700 automatic biochemistry analyzer.

### 2.13 Statistical analysis

All quantitative data are expressed as mean ± standard deviation (SD). Statistical analyses were performed using GraphPad Prism 8.0.1. One-way analysis of variance (ANOVA) with Tukey’s *post hoc* test was applied for multi-group comparisons. Statistical significance was set at P < 0.05, with * indicating P < 0.05, ** for P < 0.01, and *** for P < 0.001.

## 3 Results

**Figure 1:**
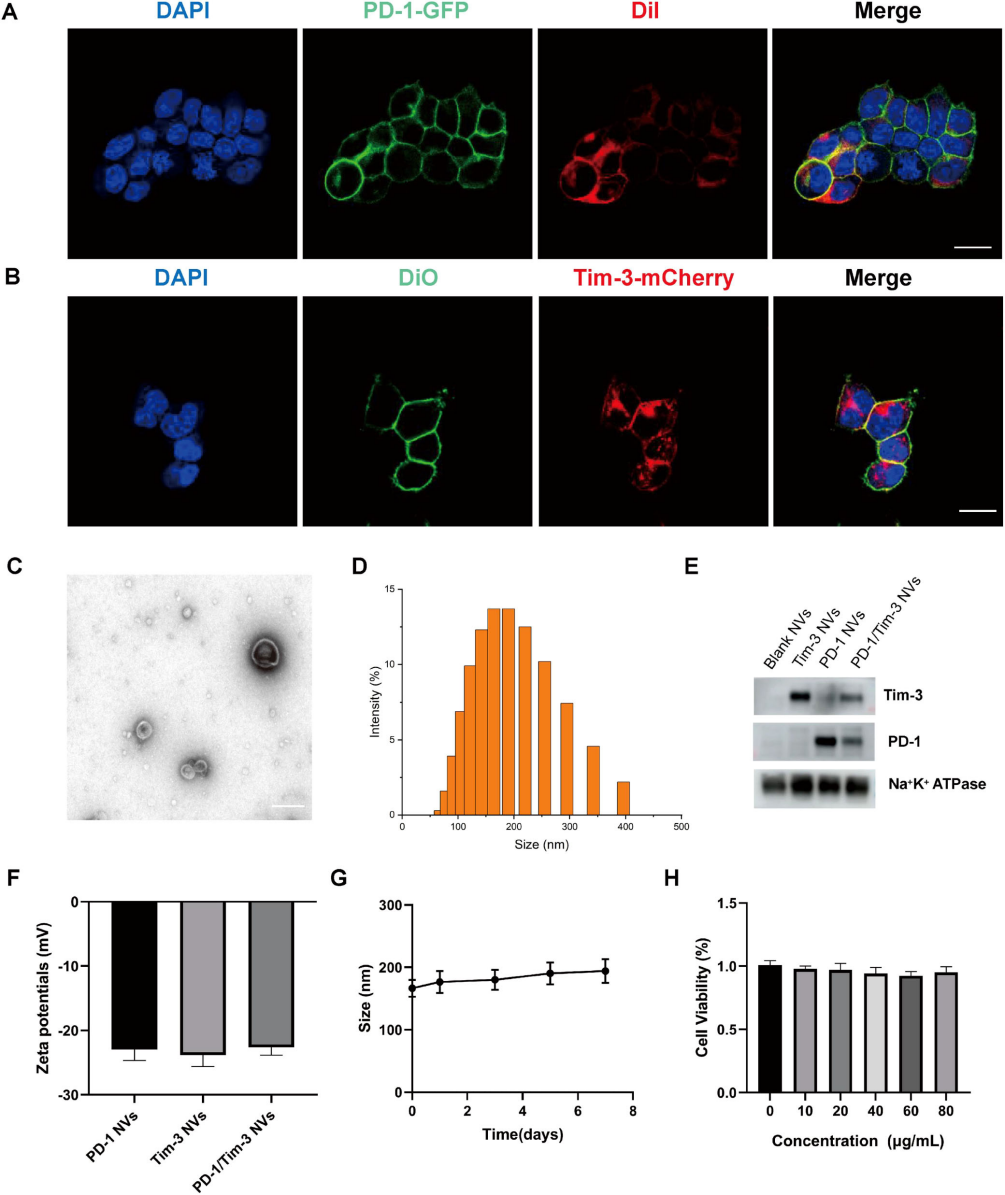
Preparation and characterization of PD-1/Tim-3 NVs. **(A)** Confocal microscopy images of PD-1-GFP stable cell line. The cell membrane was stained with membrane dyes Dil (red). Scale bar: 20 μm. **(B)** Confocal microscopy images of Tim-3-mCherry stable cell line. The cell membrane was stained with membrane dyes DiO (green). Scale bar: 20 μm. **(C)** TEM image of PD-1/Tim-3 NVs. Scale bar: 100 nm. **(D)** Size distribution of PD-1/Tim-3 NVs determined by dynamic light scattering (DLS). **(E)** Western blot analysis confirmed the retention of PD-1 and Tim-3 receptors in hybrid nanovesicles. **(F)** Zeta potential of cell membrane nanovesicles. **(G)***In vitro* stability analysis of PD-1/Tim-3 NVs. **(H)** Biocompatibility analysis of PD-1/Tim-3 NVs in HEK-293T cells via CCK-8 assay.

In a previous study, we established a stable PD-1 cell line in HEK-293T cells for the generation of PD-1 nanovesicles (27). PD-1 receptors were mainly distributed in the cell membrane of stable cells (**Figure 1A**). A stable Tim-3-expressing cell line was generated using a lentiviral vector encoding Tim-3-mCherry gene following puromycin selection (**Figure 1B**, **Supplementary Figure S1**). Co-localization of the Tim-3 protein with the plasma membrane was confirmed using the cell membrane probe Dil. Subsequently, cell membranes were isolated from the PD-1 and Tim-3 stable cell lines for nanovesicle preparation. These membranes were mixed at a 1:1 protein ratio and transformed into PD-1/Tim-3 hybrid nanovesicles (PD-1/Tim-3 NVs) via extrusion (**Supplementary Figure S2**). Transmission electron microscopy (TEM) revealed that the resulting NVs possessed a hollow spherical nanostructure (**Figure 1C**). Dynamic light scattering (DLS) analysis indicated a mean particle diameter of 182.03 nm (**Figure 1D**). SDS-PAGE analysis (**Supplementary Figure S3**) and western blotting (**Figure 1E**, **Supplementary S8**) confirmed the successful incorporation of both PD-1 and Tim-3 receptors into the nanovesicles. The surface charge of the PD-1/Tim-3 NVs was determined to be -22.6 mV (**Figure 1F**). The hybrid nanovesicles maintained their integrity in PBS under agitation for seven days (**Figure 1G**), showing good stability. Furthermore, a CCK-8 assay demonstrated that the PD-1/Tim-3 NVs had no significant effect on the viability of HEK-293 cells, indicating good biocompatibility *in vitro* (**Figure 1H**).

**Figure 2:**
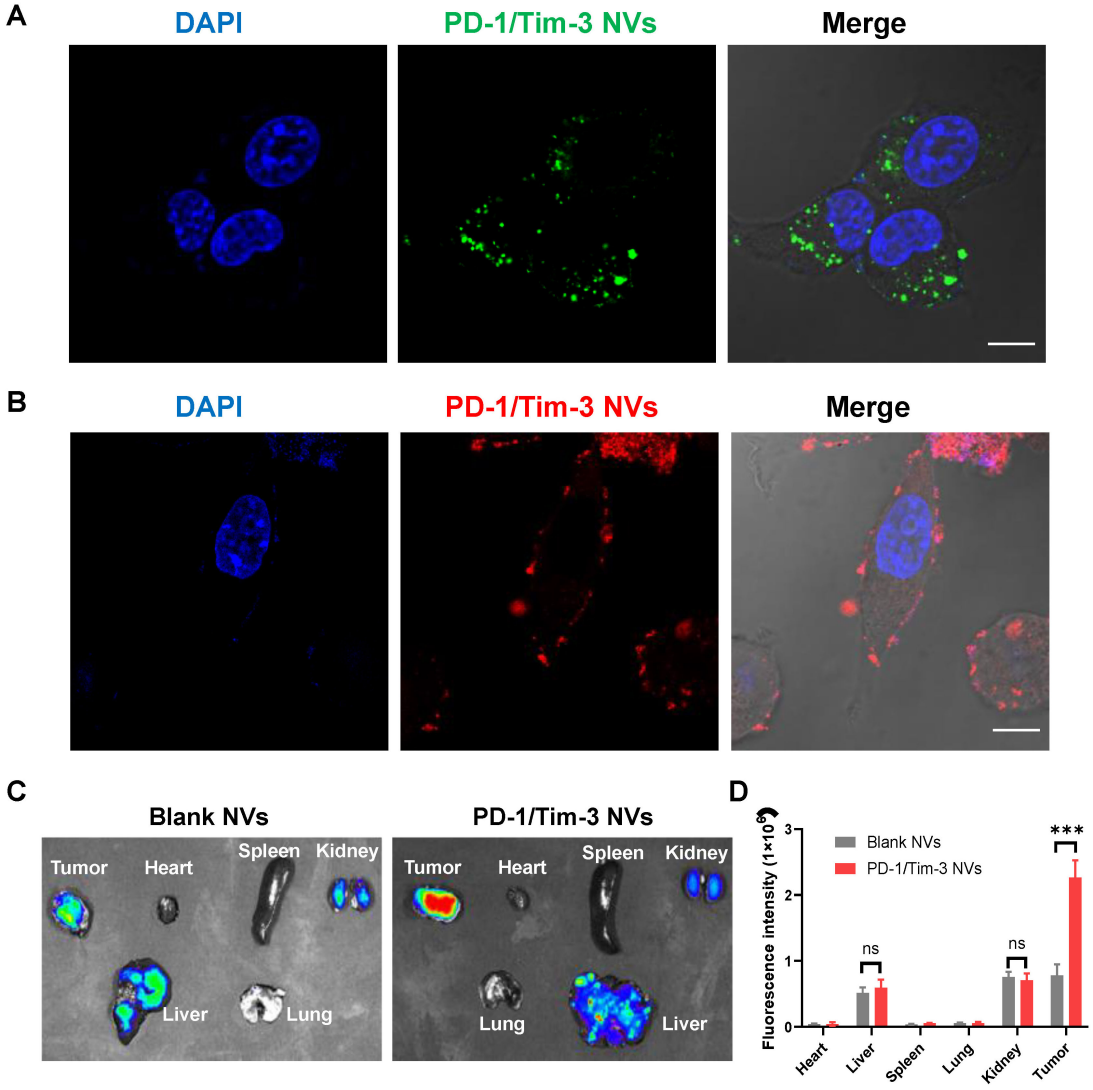
Cellular uptake and biodistribution analysis of PD-1/Tim-3 NVs. **(A)** Cellular uptake of Dio-labeled PD-1/Tim-3 NVs in CT26 cells. The nanovesicles were incubated with CT26 cells for 4h at 37 °C. Scale bar: 20 μm. **(B)** Surface binding property of PD-1/Tim-3 NVs in CT26 cells by CLSM imaging. The PD-1/Tim-3 NVs were stained with a cell membrane dye Dil (red) and incubated with CT26 cells for 1h at 37 °C. Scale bar: 20 μm. **(C)** The IVIS spectrum images of distribution of Blank NVs and PD-1/Tim-3 NVs in tumors and main organs. **(D)** Fluorescence intensity analysis of Blank NVs and PD-1/Tim-3 NVs in tumors and major organs (n = 3). ^***^P < 0.001.

The cellular uptake of PD-1/Tim-3 NVs was investigated in CT26 cells by confocal laser scanning microscopy (CLSM). CT26 cells were incubated with DiO-labeled PD-1/Tim-3 NVs for 4 hours, and bright green fluorescence localized to the perinuclear region, indicating efficient cellular uptake of the nanovesicles (**Figure 2A**). Given the presence of two immune checkpoint receptors on the nanovesicle surface, we first evaluated the selective binding of PD-1/Tim-3 nanovesicles (NVs) to CT26 tumor cells. Confocal microscopy revealed clear surface localization of DiI-labeled hybrid nanovesicles on CT26 cells, confirming specific interaction (**Figure 2B**).

To further assess *in vivo* biodistribution, we prepared Cy5.5-labeled PD-1/Tim-3 NVs and administered them intravenously into CT26 tumor-bearing mice. Fluorescence imaging using an *in vivo* imaging system (IVIS) was performed up to 24 hours post-injection to monitor accumulation in tumors and major organs. Compared with the weak tumor signal observed in mice treated with Blank NVs, PD-1/Tim-3 NVs exhibited significantly stronger tumor-associated fluorescence (**Figure 2C**), indicating enhanced tumor targeting. This improved accumulation is likely attributable to the dual decoration of PD-1 and Tim-3 receptors on the nanovesicle surface, which facilitates binding to their respective ligands (PD-L1 and galectin-9) expressed on CT26 cells (**Figure 2D**).

**Figure 3:**
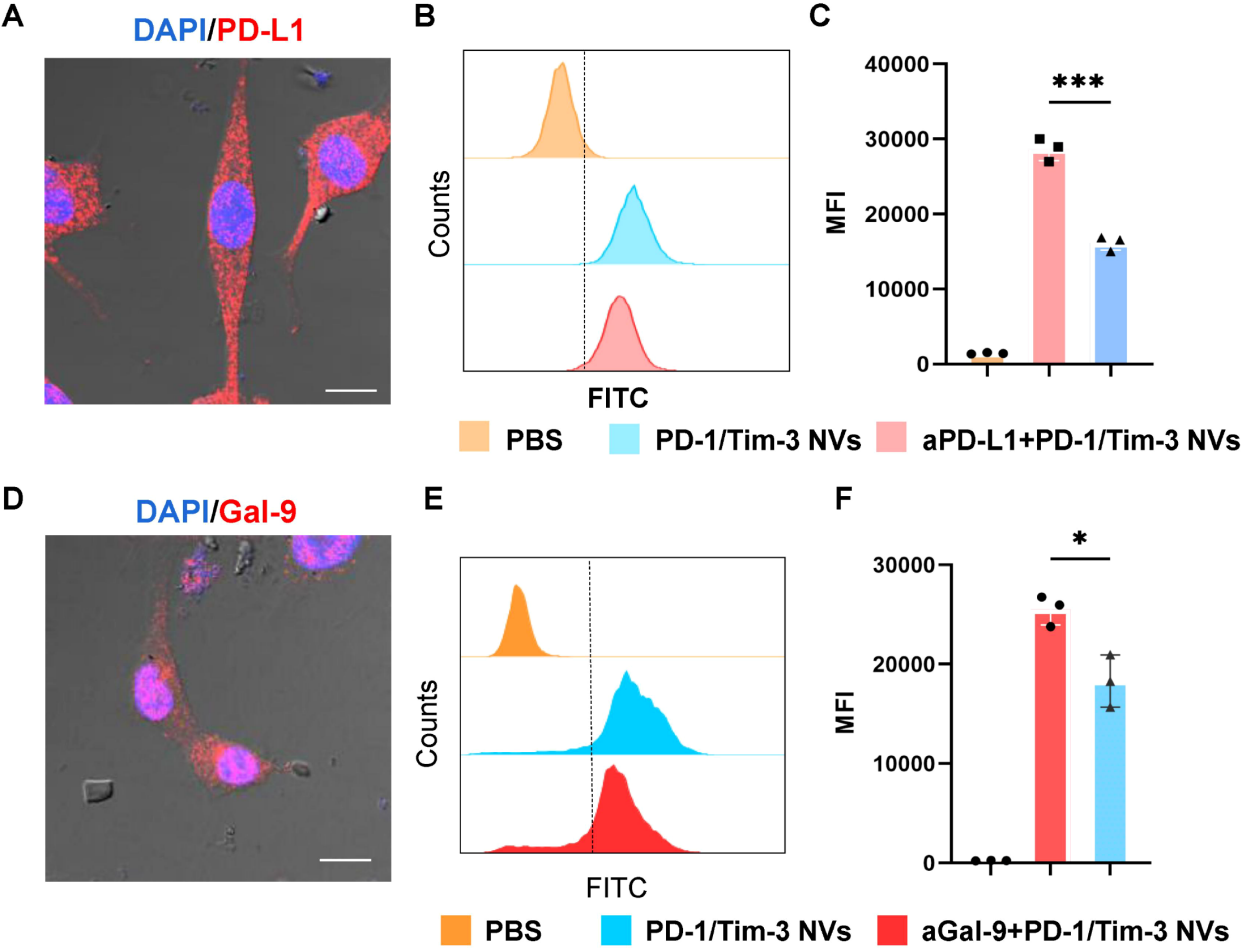
*In vitro* PD-L1 and Gal-9 analysis of PD-1/Tim-3 NVs. **(A)** Immunostaining of PD-L1 ligand in CT26 cells. **(B, C)** flow cytometry analysis and quantitative analysis of PD-L1 blockade by PD-1/Tim-3 NVs *in vitro*. **(D)** Immunostaining of Gal-9 ligand in CT26 cells. **(E, F)** flow cytometry analysis and quantitative analysis of Gal-9 blockade by PD-1/Tim-3 NVs *in vitro*. ^*^P < 0.05, ^***^P < 0.001.

Owing to the selective interaction of PD-1/PD-L1 and Tim-3/galectin-9 (36), we sought to determine if this interaction was mediated specifically by PD-1/PD-L1 and Tim-3/Gal-9 engagement. Immunostaining results demonstrated that PD-L1 was primarily localized to the plasma membrane and cytoplasmic region (**Figure 3A**). Pre-treatment of cells with an anti-PD-L1 antibody (aPD-L1) significantly inhibited the binding of PD-1/Tim-3 NVs to CT26 cells (**Figures 3B, C**). Similarly, although predominantly cytoplasmic, Gal-9 (a canonical ligand of Tim-3) is expressed in these cells (**Figure 3D**), and pre-treatment with an anti-Gal-9 antibody (aGal-9) also markedly reduced nanovesicle binding (**Figures 3E, F**). Collectively, these results demonstrate that PD-1/Tim-3 NVs selectively bind to CT26 cells *in vitro* through specific interactions with the PD-L1 and Gal-9 ligands.

**Figure 4:**
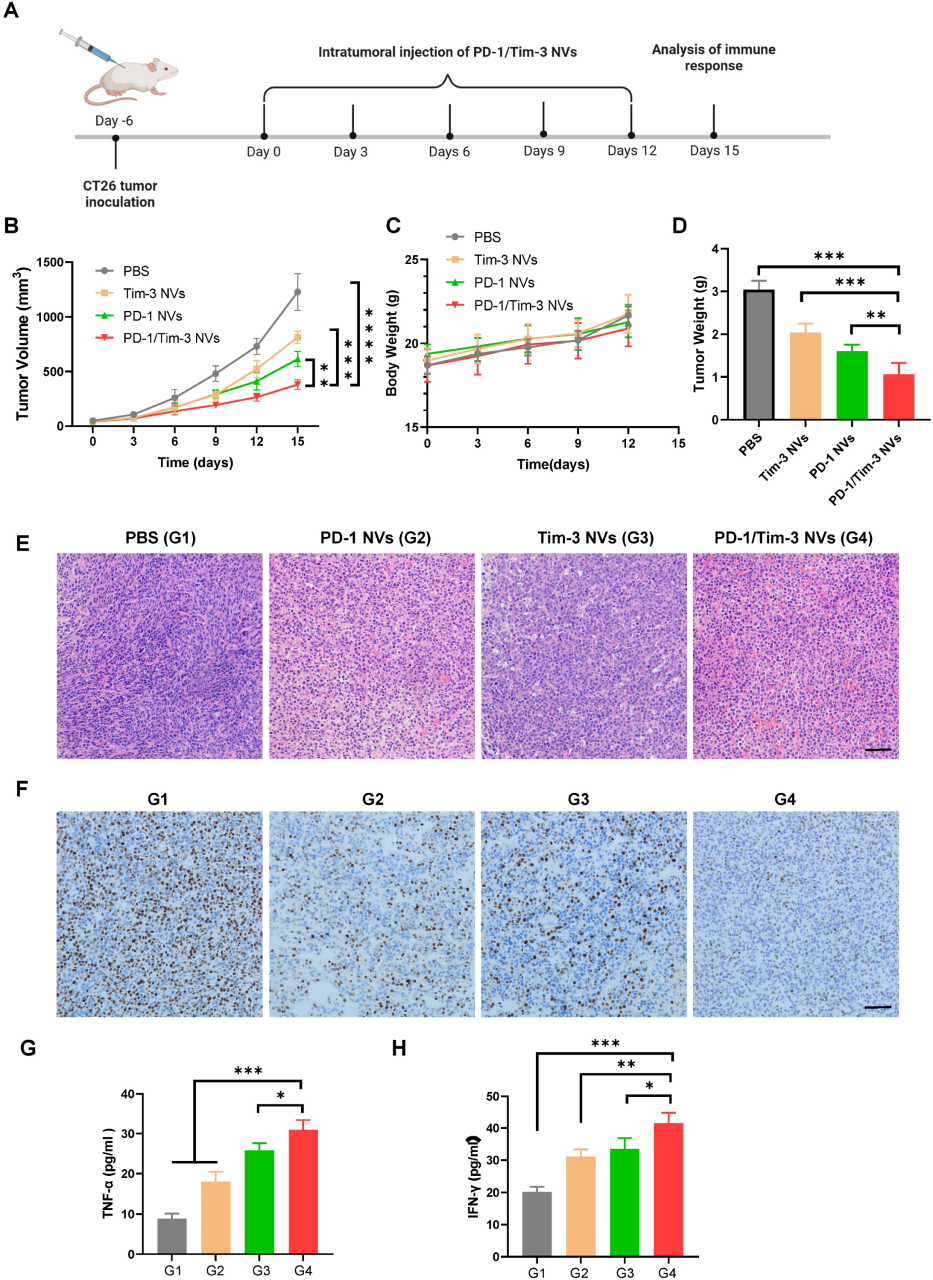
*In vivo* antitumor analysis of PD-1/Tim-3 NVs in mice bearing CT26 xenograft. **(A)** Schematic illustration of PD-1/Tim-3 NVs treatment. **(B)** Tumor growth profiles in mice treated with PBS, PD-1 NVs, Tim-3 NVs, and PD-1/Tim-3 NVs (n=6). **(C)** Body weight of mice receiving different treatments. **(D)** Excised tumor weight analysis in different groups. **(E)** H&E staining of tumor tissue sections in different groups. PD-1/Tim-3 NVs treatment significantly reduced the density of tumor cells. Scale bar: 100 μm. **(F)** Ki67 immunostaining of tumor tissues in mice treated with PBS (G1), PD-1 NVs(G2), Tim-3 NVs(G3), PD-1/Tim-3 NVs(G4). Brown nuclear staining indicates Ki67-positive proliferating cells. The PD-1/Tim-3 nanovesicle group shows a marked reduction in Ki67-positive nuclei compared to all other groups. Scale bar: 100 μm. **(G)** TNF-α level in the serum of mice treated with PBS, PD-1 NVs, Tim-3 NVs, PD-1/Tim-3 NVs. H) IFN-γ level in the serum of different groups. ^*^P < 0.05,^**^P < 0.01, ^***^P < 0.001, ****P < 0.0001.

We further evaluated the antitumor efficacy of PD-1/Tim-3 NVs in a murine CT26 xenograft model. Mice were randomly assigned to four treatment groups: PBS, PD-1 NVs, Tim-3 NVs, and PD-1/Tim-3 NVs. Based on previous studies (37, 38), each mouse received five intratumoral injections of the respective nanovesicles (0.2 mg total protein per dose) at 3-day intervals (**Figure 4A**). By day 15, treatment with PD-1 NVs, Tim-3 NVs, and the dual PD-1/Tim-3 NVs resulted in tumor inhibition rates of 33.7%, 50.1%, and 69.0%, respectively (**Figure 4B**). Among the tested three nanovesicles, the PD-1/Tim-3 NVs exhibiting the most potent suppressive effect. No significant body weight loss was observed in any group throughout the treatment period, indicating the favorable biosafety profile of the nanovesicles (**Figure 4C**). The excised tumor weights from the PD-1/Tim-3 NV group were significantly lower than those from the single-agent NV groups (**Figure 4D**).

Histological analysis further validated the good therapeutic outcome of hybrid nanovesicles. In contrast to the PBS control group, which exhibited densely packed tumor cells with little necrosis, PD-1/Tim-3 nanovesicle treatment resulted in significantly reduced tumor cellularity and increased areas of necrosis, consistent with an effective anti-tumor immune response (**Figure 4E**). To quantify tumor cell proliferation, we performed immunohistochemistry for Ki67. The PBS control group displayed a high percentage of Ki67-positive nuclei. A moderate reduction was observed in the PD-1 NVs and Tim-3 NVs groups, while the PD-1/Tim-3 NVs group showed the most profound and significant decrease in Ki67-positive cells, demonstrating a potent anti-proliferative effect (**Figure 4F**).

We also assessed the systemic inflammatory response induced by the nanovesicles by measuring secreted cytokines in serum. Levels of TNF-α and IFN-γ were quantified by ELISA across all treatment groups. Compared to the modest increase observed in single-decorated nanovesicles, PD-1/Tim-3 NVs significantly enhanced TNF-α secretion (**Figure 4G**). A similar trend was observed for IFN-γ, with the hybrid nanovesicles eliciting the strongest induction among all four groups (**Figure 4H**). Collectively, these results indicate that the PD-1/Tim-3 NVs can substantially delay tumor growth in a colorectal cancer model.

**Figure 5:**
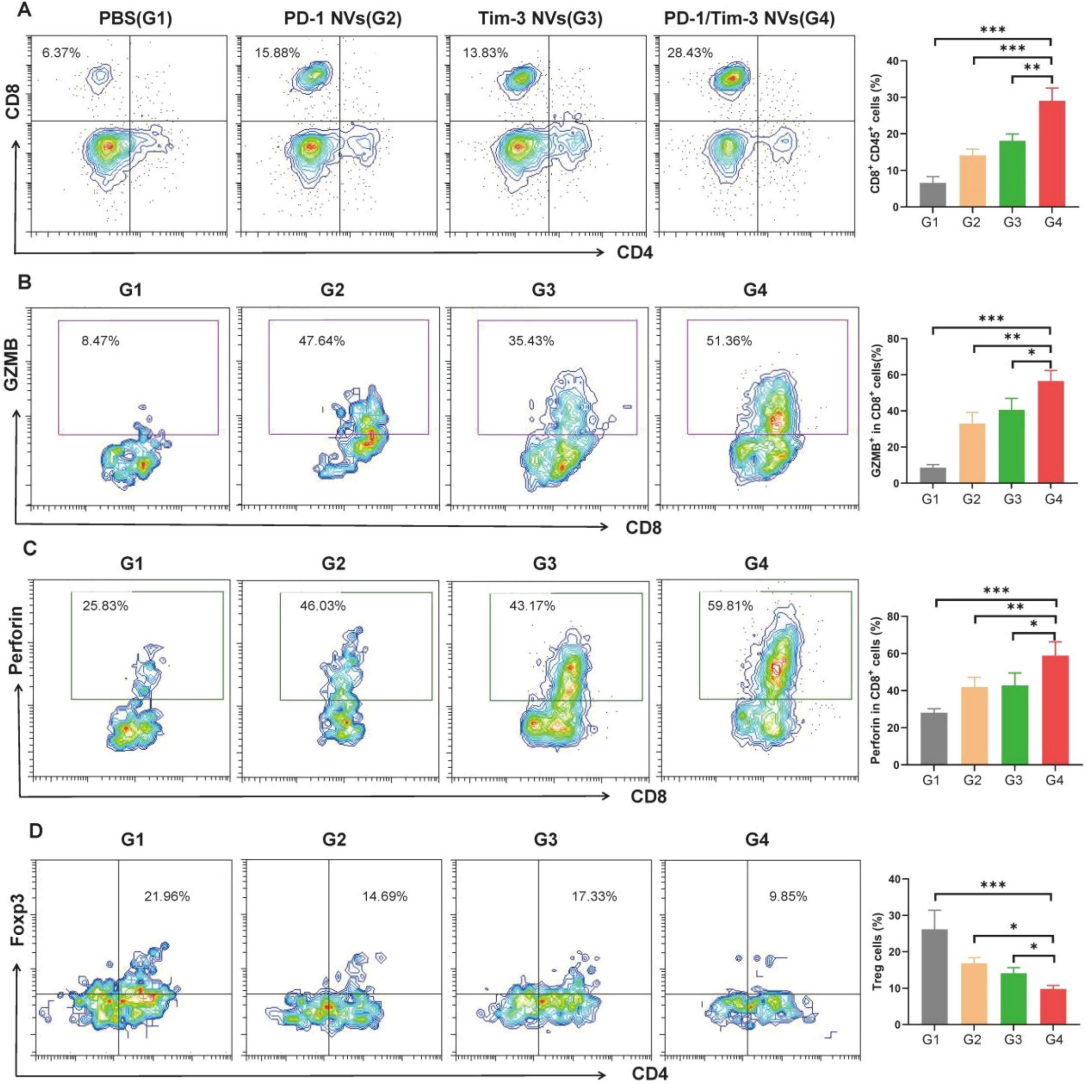
PD-1/Tim-3 NV elicited an antitumor immune response in mice bearing colorectal cancer xenografts. **(A)** Flow cytometry analysis of CD8^+^ T cell infiltration in tumors (gated on CD45^+^CD8^+^ cells). **(B)** Tumor-infiltrated GZMB-positive CD8^+^ T cells (gated on CD8^+^GZMB^+^ cells). **(C)** Flow cytometry analysis of perforin-positive CD8^+^ T cells (gated on CD8^+^perforin^+^ cells). **(D)** Tumor-infiltrated Treg cells (gated on CD4^+^Foxp3^+^ cells) by flow cytometry analysis. *P < 0.05, **P < 0.01, ***P < 0.001.

To determine whether the observed tumor suppression was mediated by antitumor immunity, we analyzed tumor-infiltrating immune cells from each group via flow cytometry. Notably, PD-1 NVs, Tim-3 NVs, and PD-1/Tim-3 NVs increased the proportion of CD8^+^ T cells within the tumor microenvironment, with the hybrid nanovesicles producing the most significant enhancement (**Figure 5A**, **Supplementary Figure S4**). We next assessed the activation status of these CD8^+^ T cells by measuring the expression of the cytolytic effector molecules perforin and granzyme B (GZMB). The frequencies of CD8^+^ GZMB^+^ (**Figure 5B**) and CD8^+^perforin^+^ T cells (**Figure 5C**) were substantially higher in the PD-1/Tim-3 NV group than in the PD-1 NVs and Tim-3 NVs groups, indicating the advantages of dual blockade in promoting T cell activation. Conversely, we evaluated the presence of immunosuppressive regulatory T cells (Tregs). Tumors from the hybrid nanovesicle group exhibited a marked decrease in Treg infiltration (**Figure 5D**, **Supplementary Figure S5**), which means that PD-1/Tim-3 NVs reprogrammed a pro-inflammatory tumor microenvironment.

Histological analysis of major organs and serum biochemical markers for hepatic and renal function showed no obvious signs of toxicity associated with PD-1/Tim-3 NV administration *in vivo* (**Supplementary Figures S6**, **S7**). Collectively, these results demonstrate that the potent antitumor efficacy of PD-1/Tim-3 NVs is driven by a coordinated immune response: enhancing cytotoxic CD8^+^ T cell infiltration and activation while simultaneously suppressing Treg-mediated immunosuppression.

## 4 Discussion

Cell-derived nanovesicles have garnered considerable attention as versatile platforms for delivering diverse therapeutic cargos in cancer and other diseases (39–41). Cell membrane nanovesicles engineered from cancer cells, bacteria, erythrocytes, platelets, macrophages, dendritic cells, and other cell types offer many advantages such as enhanced tumor targeting, prolonged circulation half-life, and reduced immune clearance for enhanced cancer therapy (42, 43). Notably, the incorporation of immune checkpoint receptors onto the nanovesicle surface endows them with immunomodulatory capabilities (44). In this study, we developed hybrid nanovesicles decorated with both PD-1 and Tim-3 to treat colorectal cancer. These dual-blockade nanovesicles simultaneously block two key inhibitory axes, thereby unleashing robust antitumor immunity and enhancing the efficacy of cancer immunotherapy.

Nevertheless, our hybrid nanovesicle platform still faces certain limitations in cancer therapy. Compared with the robust antitumor efficacy reported for combination therapy using anti-PD-1 (or anti-PD-L1) and anti-Tim-3 antibodies (45, 46), the hybrid nanovesicles in our study demonstrated relatively modest tumor suppression. This discrepancy may be attributed to the lower binding affinity between the recombinant immune checkpoint receptors displayed on the nanovesicles and their cognate ligands in the tumor microenvironment. More importantly, the suboptimal surface density of PD-1 and Tim-3 proteins on the nanovesicles present a significant challenge for clinical translation. In this work, we evaluated the antitumor activity of PD-1/Tim-3 NVs in a murine model of colorectal cancer; future studies in additional tumor types will be essential to assess the broader applicability and generalizability of this approach.

## 5 Conclusion

In this study, we engineered a hybrid nanovesicle for the dual blockade of the PD-1 and Tim-3 immune checkpoints to achieve combination immunotherapy against colorectal cancer. The resulting PD-1/Tim-3 NVs inhibited tumor growth by 69.0% in a CT26 xenograft model. The nanovesicles promoted infiltration and activation of cytotoxic CD8^+^ T cells while depleting immunosuppressive regulatory T cells. Together, our findings underscore the utility of cell membrane-derived nanovesicles as a versatile platform for dual checkpoint inhibition.

## Data Availability

The original contributions presented in the study are included in the article/**Supplementary Material**. Further inquiries can be directed to the corresponding authors.
