# Supplementary material for: Dual checkpoint blockade of PD-1 and Tim-3 by engineered hybrid nanovesicles for enhanced cancer immunotherapy
Source: Front Immunol. 2025 Dec 17;16:1705438. doi: 10.3389/fimmu.2025.1705438 (PMC12753386; doi:10.3389/fimmu.2025.1705438)
Supplement: Supplementary file 1 [file DataSheet1.docx]

**Dual Checkpoint Blockade of PD-1 and Tim-3 by Engineered Hybrid Nanovesicles for Enhanced Cancer Immunotherapy**

Han Xue^1,2#^, Longxue Guan^2#^, Lili Huang ^2#^, Yuxin Fan^2^, Fenglin Guo^2^, Dandan Liang^2^, Xingang Guan^1,*^ Guofu Chen^1,*^

^1^ The First People’s Hospital of Wenling (Taizhou University Affiliated Wenling Hospital), School of Medicine, Taizhou University, Taizhou 317500, PR China

^2^ College of Medical Technology, Beihua University, Jilin 132013, PR China

**Corresponding authors:** guanxg@ciac.ac.cn (X. Guan), wlcgf@163.com (G. Chen).

^#^ These authors contribute to this work equally.


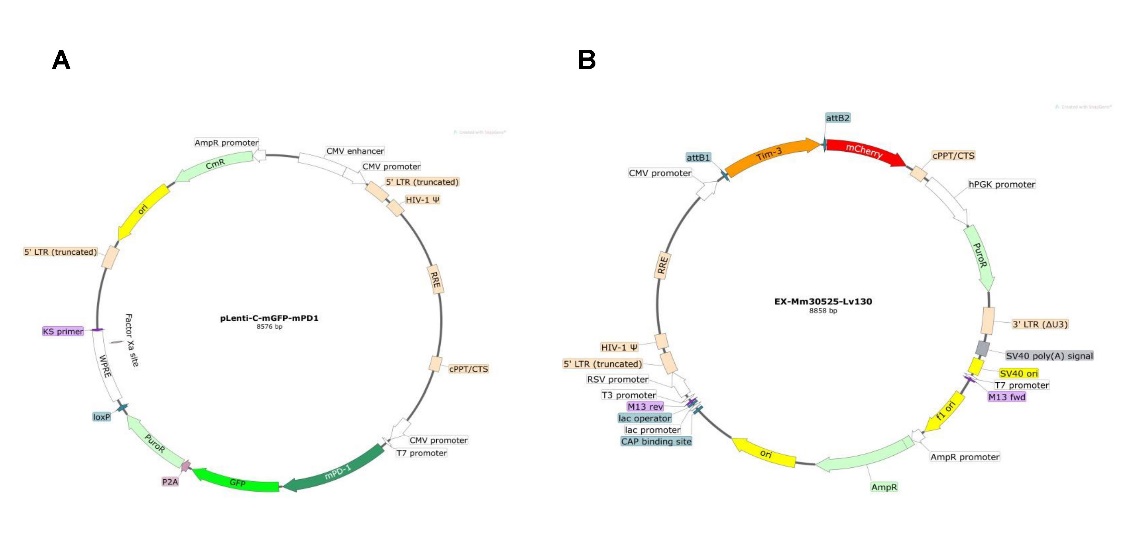


**Figure S1.** Vector map of pLenti-C- PD-1-mGFP and pEZ-Lv130-Tim-3-mCherry.


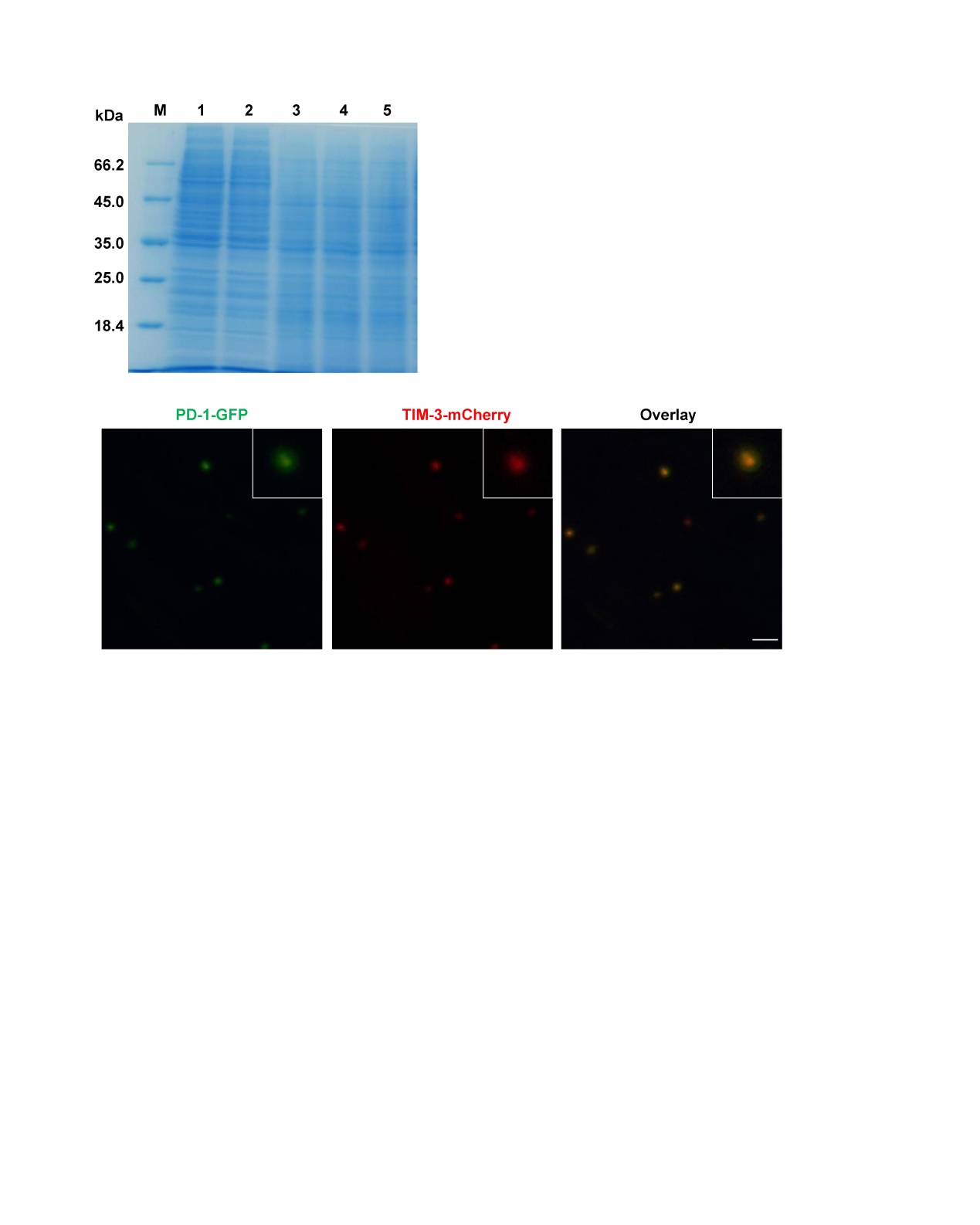


**Figure S2.** Fluorescent imaging of PD-1/Tim-3 NVs. The merged yellow sphere demonstrated the coexistence of PD-1-GFP and Tim-3-mCherry on the surface of hybrid NVs. Scale bar: 10 μm.


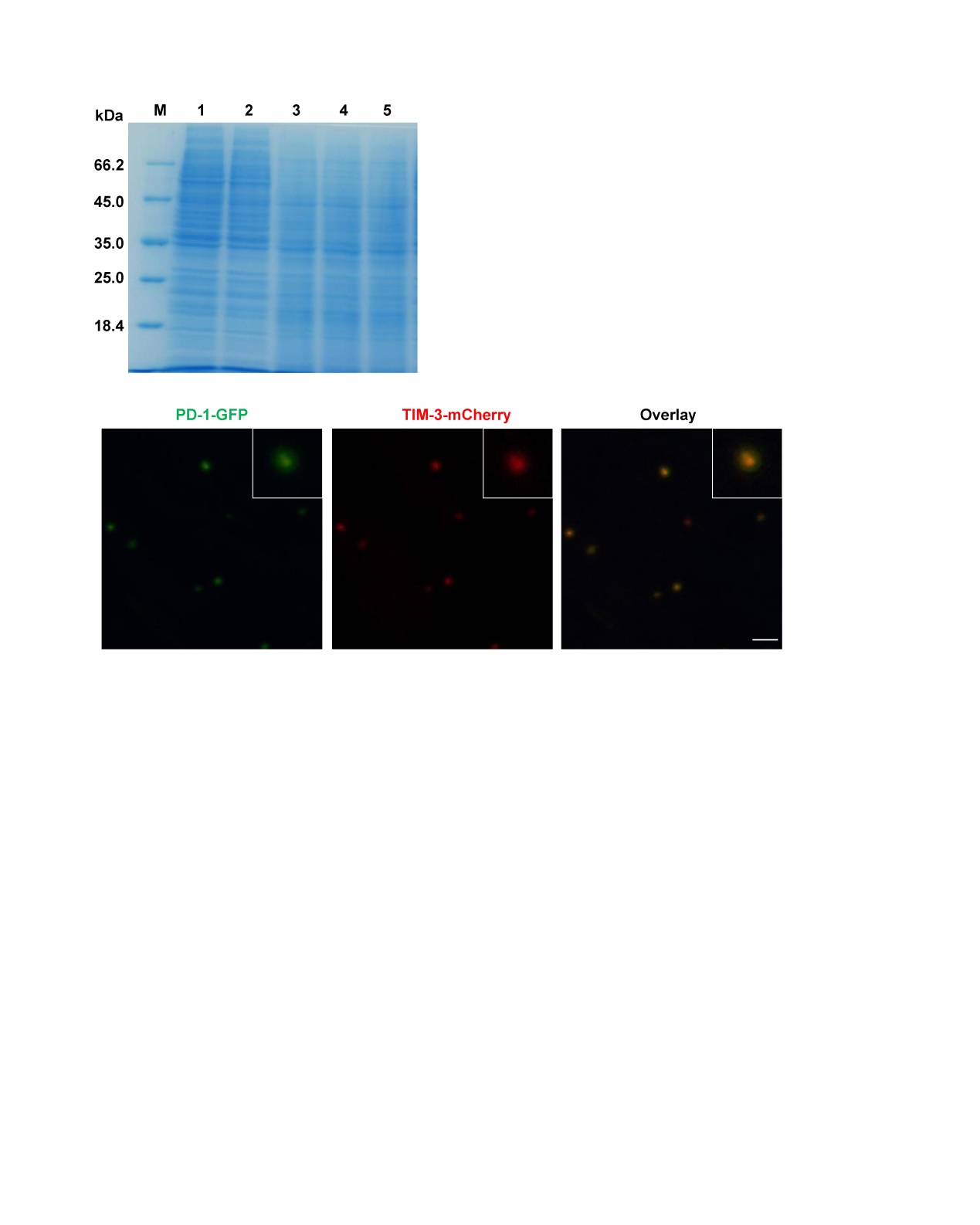


**Figure S3.** SDS-PAGE analysis of PD-1/Tim-3 NVs. Lane 1: PD-1 stable cells; Lane 2: Tim-3 stable cells; Lane 3: PD-1 NVs; Lane 4: Tim-3 NVs; Lane 5: PD-1/ Tim-3 NVs.


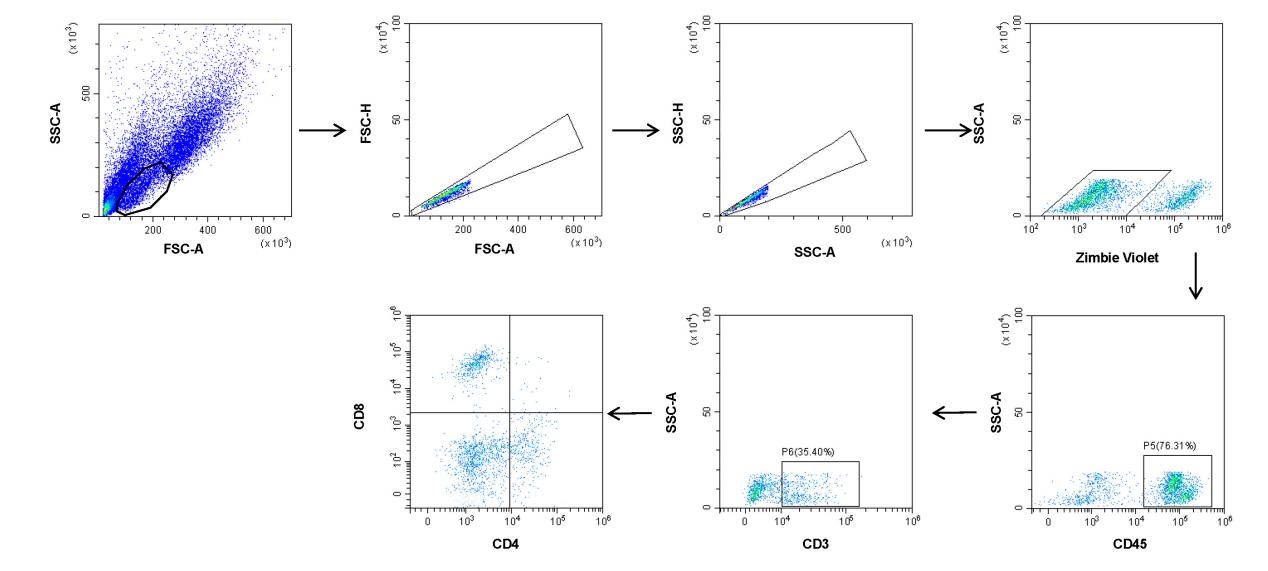


**Figure S4.** Flow cytometry gating strategies for CD8^+^ T cells in tumors. The cells were gated for positive CD45^+^CD8^+^ expression.


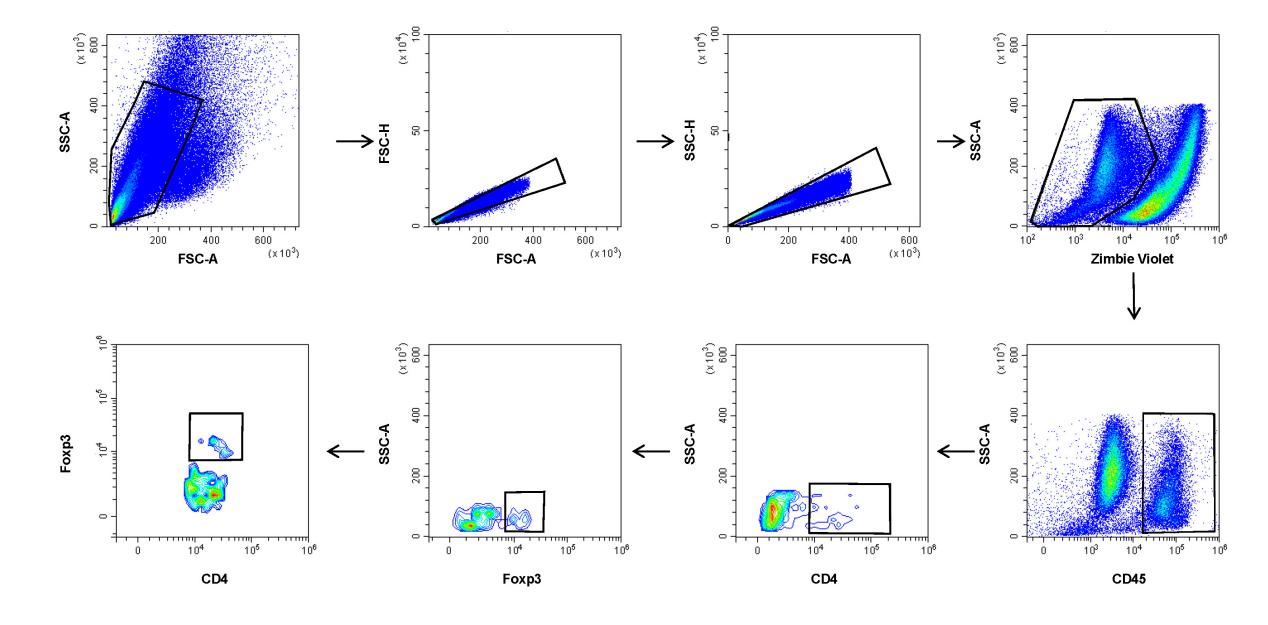


**Figure S5**. Flow cytometry gating strategies for Treg cells in tumors. The cells were gated for positive CD45^+^CD4^+^Foxp3^+^ expression.


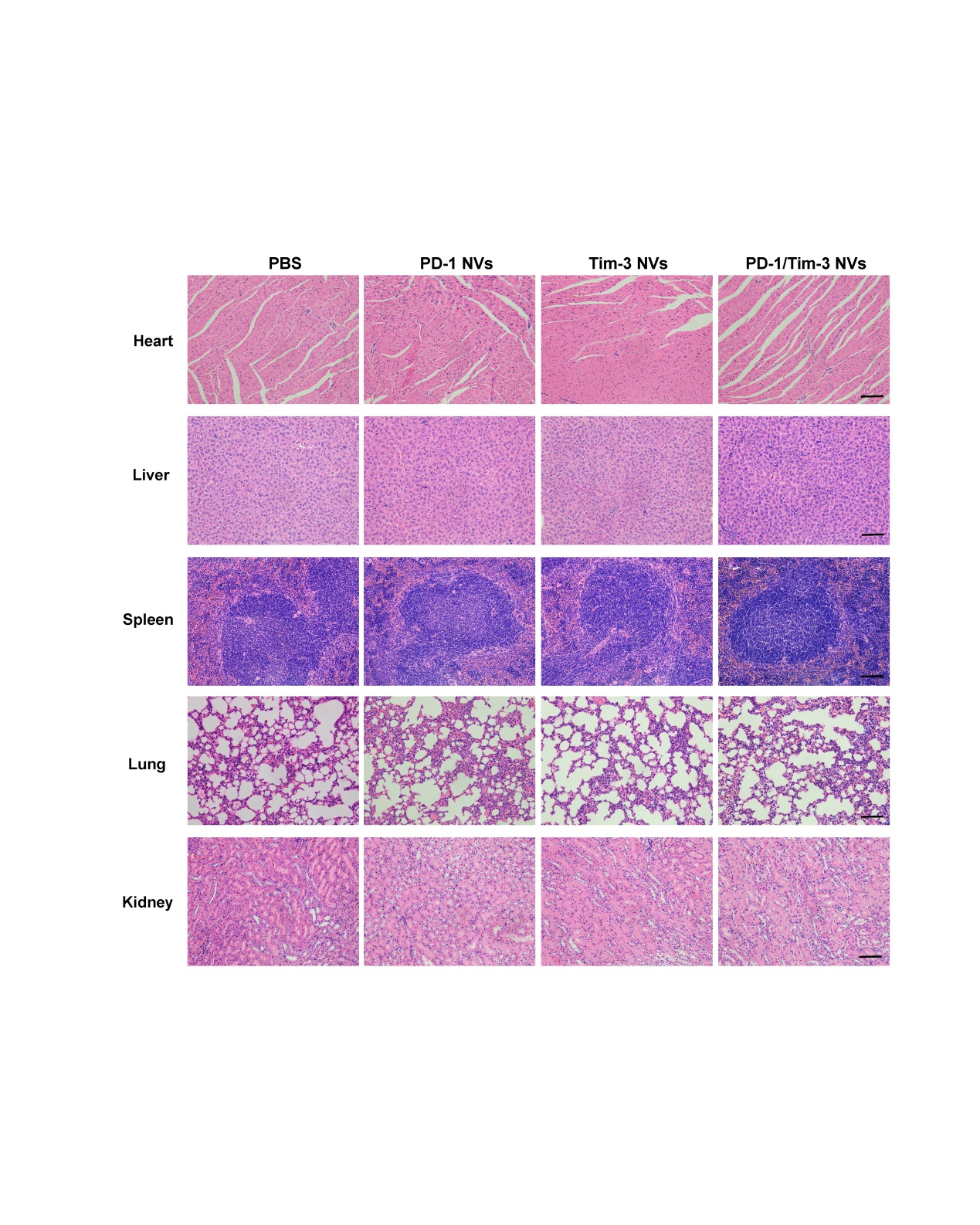


**Figure S6.** Histological images for H&E staining obtained from the heart, liver, spleen, lung, and kidney of mice with different treatments. Scale bar: 100 µm.


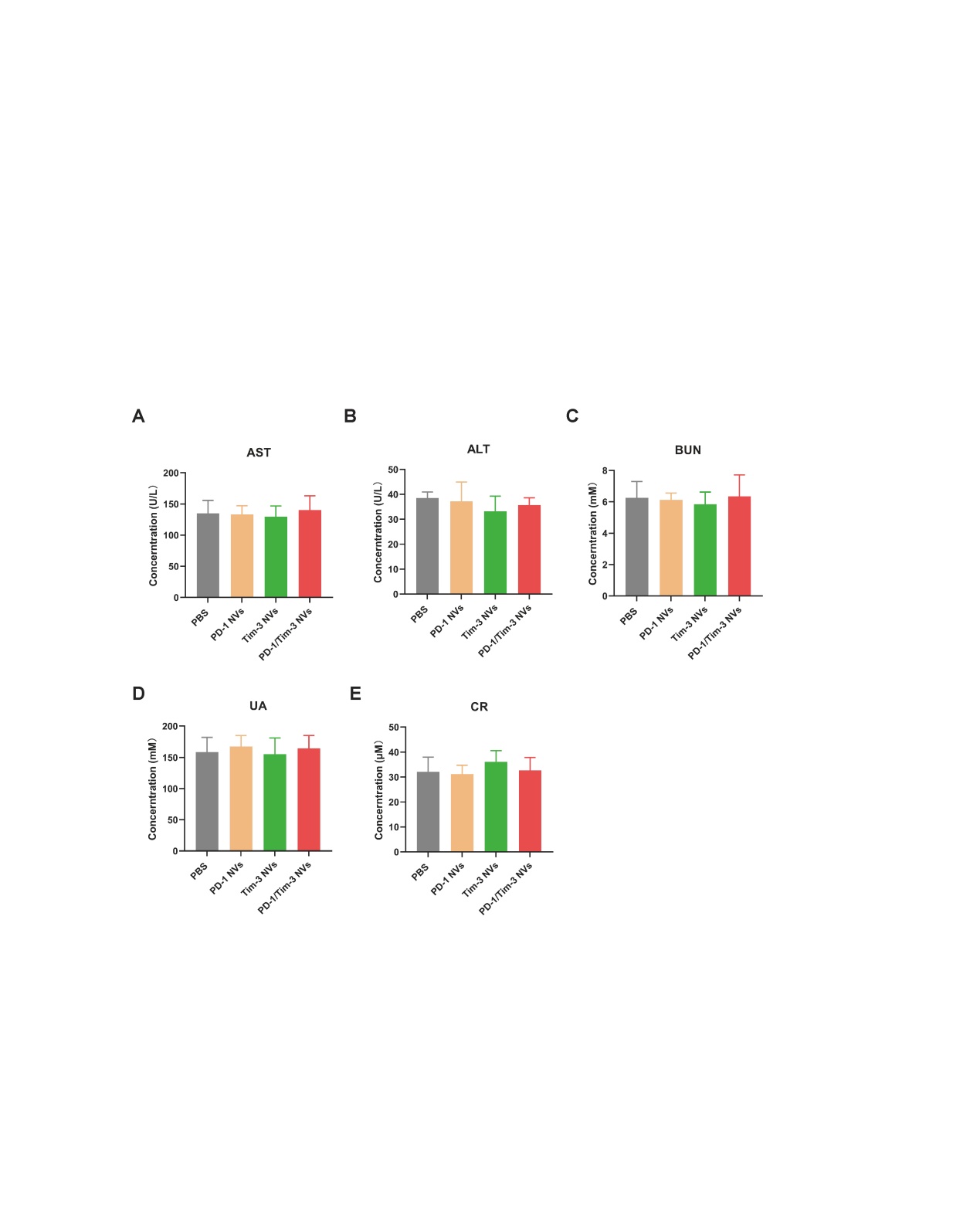


**Figure S7**. Serum biochemical analysis of kidney and liver function parameters. Data are expressed as mean ± SD (n=3).


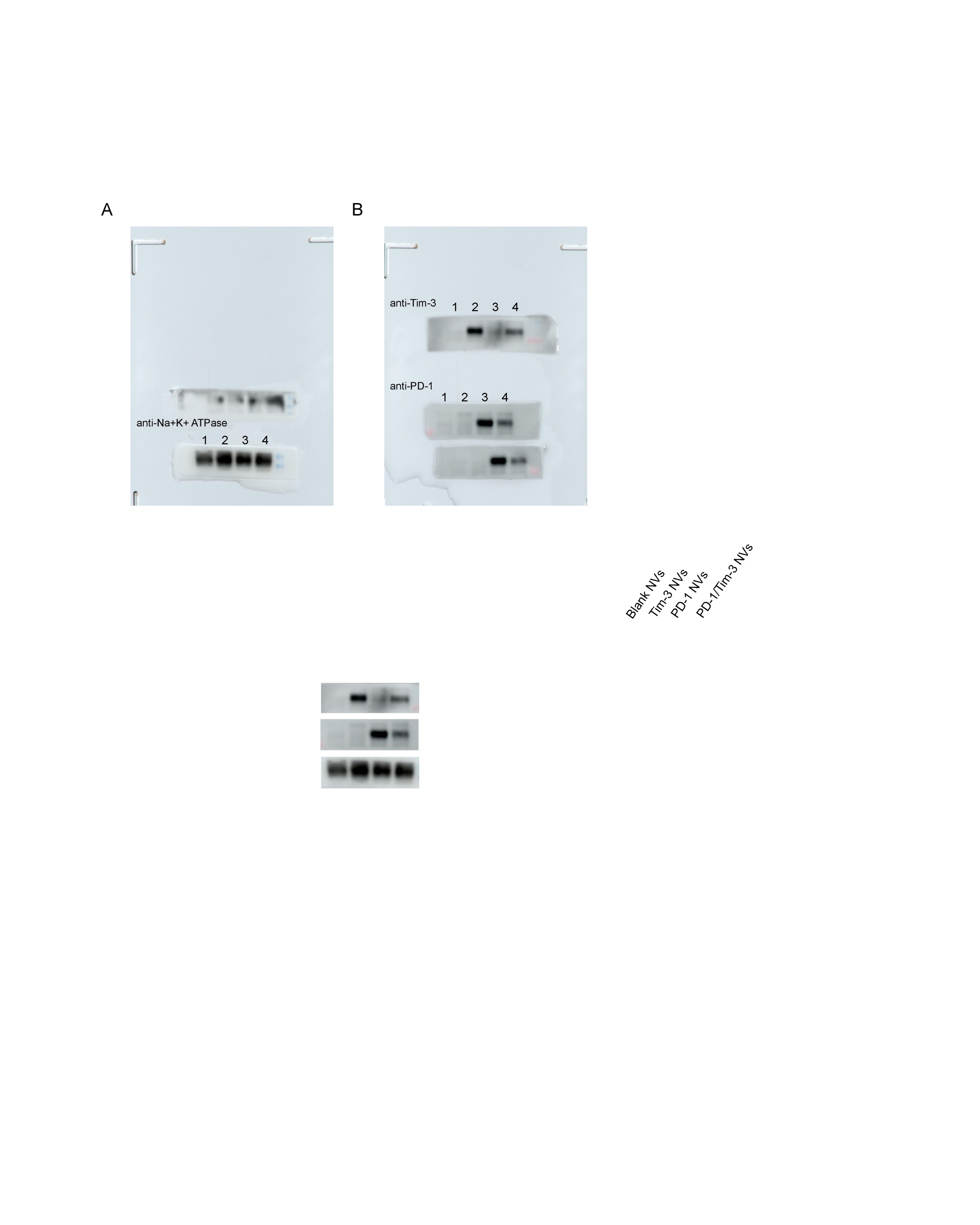


**Figure S8.** Uncropped Western blot images corresponding to Figure 1E. (A) Detection of Na⁺/K⁺ ATPase. (B) Detection of Tim-3 (upper panel) and PD-1 (lower panel). Lane 1: Blank NVs; Lane 2: Tim-3 NVs; Lane 3:PD-1 NVs; Lane 4: PD-1/ Tim-3 NVs
